# Educational Case: Lambert–Eaton syndrome

**DOI:** 10.1016/j.acpath.2022.100021

**Published:** 2022-05-12

**Authors:** Austin Huddleston, Larry Nichols

**Affiliations:** Mercer University School of Medicine, Macon, GA, USA

**Keywords:** Pathology competencies, Organ system pathology, Nervous system-peripheral nervous system and eye, Peripheral nerve disorders, Lambert–Eaton syndrome, Myasthenia gravis, Small-cell carcinoma, Hypersensitivity

The following fictional case is intended as a learning tool within the Pathology Competencies for Medical Education (PCME), a set of national standards for teaching pathology. These are divided into three basic competencies: Disease Mechanisms and Processes, Organ System Pathology, and Diagnostic Medicine and Therapeutic Pathology. For additional information, and a full list of learning objectives for all three competencies, see https://www.journals.elsevier.com/academic-pathology/news/pathology-competencies-for-medical-education-pcme.^1^

## Primary objective

Objective NSP1.1: Neuromuscular Junction disorders. Describe the clinicopathologic features of antibody-mediated disorders of the neuromuscular junction such as myasthenia gravis and Lambert–Eaton myasthenic Syndrome.

Competency 2: Organ system pathology; Topic: Nervous system—Peripheral nervous system and eye (NSP); Learning goal 1: Peripheral nerve disorders.

## Secondary objective

Objective IM1.4: Hypersensitivity. Compare and contrast the mechanisms of the 4 hypersensitivity reactions with respect to the situations in which each is triggered, mechanisms of injury, resulting pathologic effects on tissue, and the ultimate clinical consequences.

Competency 1: Disease mechanisms and processes; Topic: Immunological mechanisms (IM); Learning goal 1: Immune dysfunction.

## Patient presentation

A 60-year-old man presents with muscle weakness that started approximately 18 months ago and has gradually progressed. The weakness was first evident getting up from bed or a chair but progressed to difficulty climbing stairs. Ten months after symptom onset, the patient was evaluated by nerve conduction tests, which were nondiagnostic. Routine chest radiograph showed findings of pulmonary emphysema. Eight months later, he now presents with generalized muscle weakness and fatigue for the past month or so. On questioning, he describes the weakness as constant, and denies noticing anything that makes the weakness better or worse. He denies significant past medical history. He is on no medications. He lives a sedentary lifestyle. He has smoked 1 pack of cigarettes per day for 42 years, has drunk 6 bottles of beer per day for many years, and denies using illicit drugs. On review of systems, the patient reports erectile dysfunction for the past several months. He also has mild dryness of the eyes and mouth. The patient has had a 20-pound weight loss during the past 3 months. He denies significant muscle pain, chest pain, cough, dyspnea or skin rash.

## Diagnostic findings, Part 1

On examination, the patient's temperature is 98.6 ​°F, heart rate 75 per min, blood pressure 138/78 ​mm Hg, respiratory rate 18 min, and oxygen saturation 95% on room air. He is alert and oriented to person, place, and time. The patient has mild weakness of neck flexor and shoulder girdle muscles and moderate weakness of pelvic girdle muscles. Muscle strength increases after a sustained 30-s contraction. Deep-tendon reflexes are decreased in the lower extremities (1+ bilaterally) compared with the upper extremities (2+ bilaterally). Sensation to light touch and pinprick is intact, and Babinski's sign is down-going bilaterally. There are no cerebellar signs. Cranial nerve examination is normal. The chest is clear to auscultation. The remainder of the physical examination is unremarkable. Blood testing shows the results in Table 1.

**Table 1:** Blood test results.

Blood test	Patient	Reference range
Hemoglobin	13 ​g/dL	13.5–17.5 ​g/dL
White blood cell count ​+ ​differential	10,500/cu mm	4500–11,000/cu mm
	60% neutrophils	40–60% neutrophils
	30% lymphocytes	20–40% lymphocytes
	10% monocytes	2–8% monocytes
Platelets	200,000/cu mm	150,000–450,000/cu mm
Glucose	105 ​mg/dL	70–99 ​mg/dL
Sodium	135 mEq/L	135–145 mEq/L
Potassium	3.9 mEq/L	3.5–5.0 mEq/L
Chloride	98 mEq/L	95–105 mEq/L
Bicarbonate	27 mEq/L	22–28 mEq/L
Blood urea nitrogen	20 ​mg/dL	6–24 ​mg/dL
Creatinine	1.1 ​mg/dL	0.74–1.35 ​mg/dL
Bilirubin	0.9 ​mg/dL	0.1–1.2 ​mg/dL
Alkaline phosphatase	69 U/L	44–147 U/L
Alanine aminotransferase	35 U/L	4–36 U/L
Aspartate aminotransferase	70 U/L	8–33 U/L

## Questions/discussion points, Part 1

### What is the differential diagnosis of proximal muscle weakness?

The differential diagnosis of proximal muscle weakness in adults includes hypokalemia, dermatomyositis, polymyositis, immune-mediated necrotizing myopathy, statin therapy, alcoholic myopathy, thyroid myopathy, myasthenia gravis, and Lambert–Eaton syndrome. Some of these conditions have associated symptoms that can focus the differential diagnosis. Hypokalemia can be from vomiting, although it is more often due to medications, particularly diuretics.^2^ Dermatomyositis has associated skin rash.^3^ Immune-mediated necrotizing myopathy is associated with myalgias and is most often due to statin therapy.^4^ Hypothyroidism can cause muscle weakness, most often in association with myalgias, muscle cramps, fatigue, hair loss, thickening skin, edema, weight gain, and other symptoms; slowed deep tendon reflexes can be a distinctive sign of hypothyroidism.^5^ Myasthenia gravis typically presents with ocular symptoms such as diplopia and ptosis, and is not associated with diminished deep tendon reflexes.^6^ Lambert–Eaton syndrome has a classic triad of clinical manifestations: proximal muscle weakness, decreased tendon reflexes, and autonomic dysfunction.^7^

### What is the most likely diagnosis in this patient based on his clinical presentation?

This patient has proximal muscle weakness and decreased deep tendon reflexes in the lower extremities. He has increased muscle strength after sustained contraction, which favors a diagnosis of Lambert–Eaton syndrome. He is on no medications and does not have hypokalemia. On questioning, he reports erectile dysfunction for the past several months, and mild dryness of the eyes and mouth, which can be symptoms of autonomic dysfunction. Lambert–Eaton syndrome is at the top of the differential diagnosis for this patient.

### How is Lambert–Eaton syndrome diagnosed?

A three-fold diagnostic process of physical examination, nerve conduction studies±electromyography, and serology is used to confirm the diagnosis of Lambert–Eaton syndrome.^6^

## Diagnostic findings, Part 2

Nerve conduction tests show severely reduced compound muscle action potential (CMAP) amplitude in the lower extremities and mildly to moderately reduced CMAP amplitude in the upper extremities. CMAP amplitude increases abnormally after 10 ​s of exercise; and CMAP amplitude increases 90%–100% with high frequency repetitive nerve-stimulation studies. Blood test for antibodies to voltage-gated calcium channels (VGCCs) return positive, confirming the diagnosis of Lambert–Eaton syndrome.

## Questions/discussion points, Part 2

### What is the interpretation of the nerve conduction tests?

CMAP amplitude is determined by a summation of all action potentials that occur at stimulated motor endplates.^8^ Decreased CMAP amplitude at rest in this patient correlates with a decreased amount of summated action potentials at the neuromuscular junctions at rest. High frequency repetitive-nerve stimulation increased CMAP amplitude by 90%–100% and indicates an increased summation of action potentials at the motor endplate.

Nerve conduction tests can be complemented by electromyography (EMG).^9^ EMG utilizes a needle electrode inserted into muscle to record the electrical activity of the muscle.^10^ Interpretation of the firing rate and various waveforms of motor unit electrical patterns can help determine if weakness is myopathic, neuropathic, or secondary to dysfunction of the neuromuscular junction.^10^ Lambert–Eaton syndrome is a disease of the nerve side of the neuromuscular junction. If EMG were performed in this patient, it may have shown a pattern of denervation or, more likely, have been normal.

### What is the pathophysiology of Lambert–Eaton syndrome?

Lambert–Eaton syndrome is a disease of the neuromuscular junction. Under normal physiological conditions, depolarization of the neuronal presynaptic membrane induces voltage-gated calcium channels (VGCCs) to open, promoting the influx of calcium into the nerve terminal. Increased calcium levels in the presynaptic nerve terminal facilitate the release of acetylcholine (ACh) into the synapse. ACh diffuses across the synapse to bind to ACh receptors postsynaptically on the muscle end-plate. This binding opens postsynaptic ligand-gated sodium and potassium channels and depolarizes the motor end-plate. After the depolarization threshold is met, an action potential occurs and muscle contraction takes place.^7^

Autoantibodies to the presynaptic VGCCs are produced in Lambert–Eaton syndrome. This results in a decrease in the amount of ACh released from the presynaptic nerve terminal.^6^ The decreased quantity of presynaptic ACh released translates to an under-activation of postsynaptic ligand-gated sodium and potassium channels at the motor end-plate. Decreased end-plate action potential is a direct result of this reduction of ion channel activation.^7^ This mechanism accounts for the muscular weakness and autonomic symptoms that are present in Lambert–Eaton syndrome.

A hallmark feature of Lambert–Eaton syndrome is the improvement of muscle weakness and deep-tendon reflexes with repeated muscle contraction. Repeated stimulation allows for sufficient amounts of ACh to be released and correlates with increased activation of postsynaptic ion channels, improving muscle weakness and tendon reflexes. This finding is evident on nerve conduction studies with a 100% increase in CMAP following brief periods of exercise being considered specific for Lambert–Eaton syndrome.^6^ It should be noted that not all patients with Lambert–Eaton syndrome will exhibit the classical improvement in muscular weakness and deep-tendon reflexes following a brief exercise period.

### Does Lambert–Eaton syndrome fit in any categories of immunologic disease?

Many antibody-mediated immune diseases are classified as type II hypersensitivity reactions. Lambert–Eaton syndrome is an immunologic antibody-mediated disease. The antibodies are directed against a self-antigen, so the first broad category of disease it fits into is autoimmune diseases. Hypersensitivity reactions are injurious immunologic responses responsible for the pathology-associated with immunologic diseases.^11^ There are four types. Type I reactions are mediated by IgE antibodies produced in response to environmental proteins such as pollens, animal danders or dust mites. Type II reactions are mediated by IgG and IgM antibodies against proteins of cell surface and extracellular matrix, which damages cells by activating the complement system or by phagocytosis. Type III reactions are mediated by IgM and IgG antibodies that bind soluble antigens forming antigen–antibody complexes and activate the complement system. Type IV reactions are mediated by T lymphocytes that provoke a delayed, sometimes granulomatous inflammatory reaction. While Lambert–Eaton syndrome superficially resembles a type II hypersensitivity reaction, it lacks the cytotoxicity, complement activation or phagocytosis characteristic of type II reactions.

### How can Lambert–Eaton myasthenic syndrome be differentiated from myasthenia gravis?

Myasthenia gravis is the disease most often at the top of the differential diagnosis in a patient who presents with Lambert–Eaton myasthenic syndrome.^6^ The incidence of myasthenia gravis is approximately ten times higher than the Lambert–Eaton myasthenic syndrome.^7^ Myasthenia gravis is an immunologic antibody-mediated disease of the neuromuscular junction like Lambert–Eaton syndrome, but due to antibodies against the ACh receptors on the motor end-plate. Lambert–Eaton syndrome typically starts with leg weakness which progresses upward, while myasthenia gravis typically begins with oculobulbar weakness and spreads downward.^6^ Autonomic dysfunction and decreased tendon reflexes are rarely seen with myasthenia gravis. Proximal leg muscles are typically the worst affected in Lambert–Eaton syndrome, while extraocular muscles are typically most involved in myasthenia gravis.^12^ With electromyographic studies in patients with Lambert–Eaton syndrome, high-frequency repetitive nerve stimulation typically yields an incremental response of 100% or greater in CMAP amplitude, but any response over 60% is considered diagnostic.^7^ Myasthenia gravis does not show such a response to repetitive nerve stimulation. Antibodies to the presynaptic VGCCs are also not seen in myasthenia gravis. Differences that separate Lambert–Eaton syndrome from myasthenia gravis are summarized in Table 2.

**Table 2:** Lambert–Eaton syndrome versus myasthenia gravis.

	Lambert–Eaton syndrome	Myasthenia gravis
Typical first symptom	Difficulty arising from bed or chair	Diplopia
Autonomic symptom	Dry mouth	None
Weakest muscles	Proximal leg	Extraocular
Deep tendon reflexes	Decreased	Normal
High frequency repetitive nerve stimulation	>60% increase in muscle action potential	<60%
Autoantibody target	Presynaptic voltage-gated calcium channel	Postsynaptic acetylcholine receptor

### For what associated disease should a diagnosis of Lambert–Eaton syndrome prompt a search?

An underlying neoplasm is present in 47%–62% of cases of confirmed Lambert–Eaton syndrome.^6^ Small-cell lung carcinoma is the most common neoplasm associated with the syndrome. Symptoms of Lambert–Eaton syndrome nearly always precede a diagnosis of small-cell lung carcinoma. A confirmed diagnosis of Lambert–Eaton syndrome should prompt screening for an associated tumor. Computerized tomography (CT) of the thorax is recommended for preliminary screening, followed by positron emission tomography (PET) if initial studies are negative. It is recommended that patients with a confirmed diagnosis of Lambert–Eaton syndrome and an initial negative oncological screening should be screened every three to six months for at least two years.^6^

## Diagnostic findings, Part 3

**Fig. 1:**
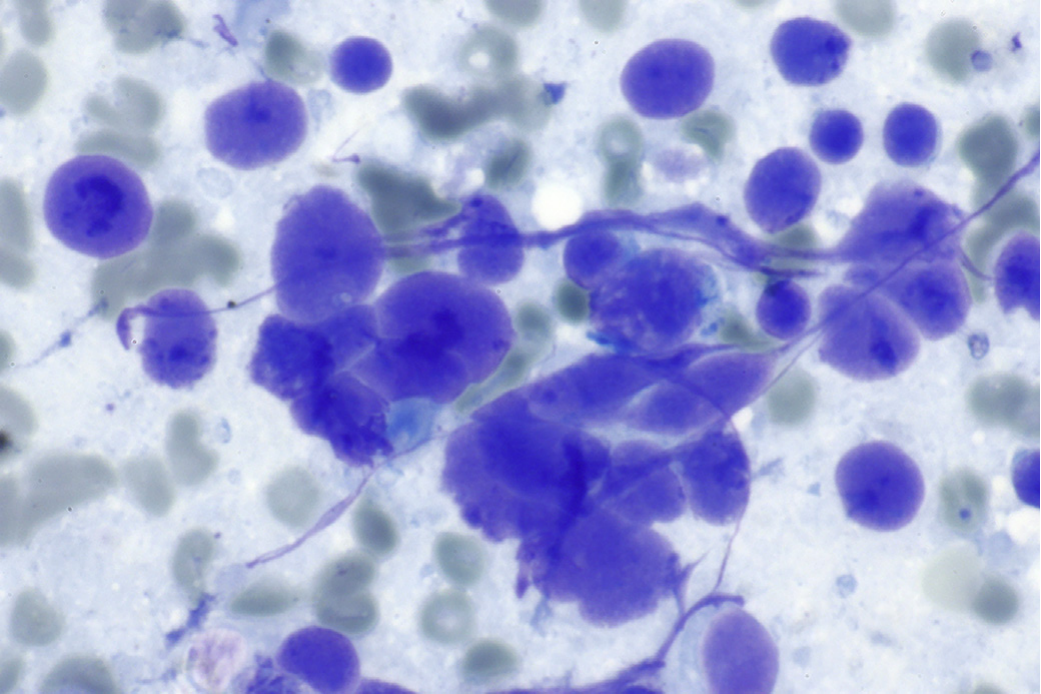
Cytological examination shows dark blue small-cell carcinoma cells 2–4 x size of gray-greenish erythrocytes in the background. (Diff-Quik stain used for rapid on site evaluation, x100) Image By Nephron – Own work, CC BY-SA 3.0, https://commons.wikimedia.org/w/index.php?curid=48340536. Republished under the Creative Commons Attribution (CC-BY-SA 3.0).

**Fig. 2:**
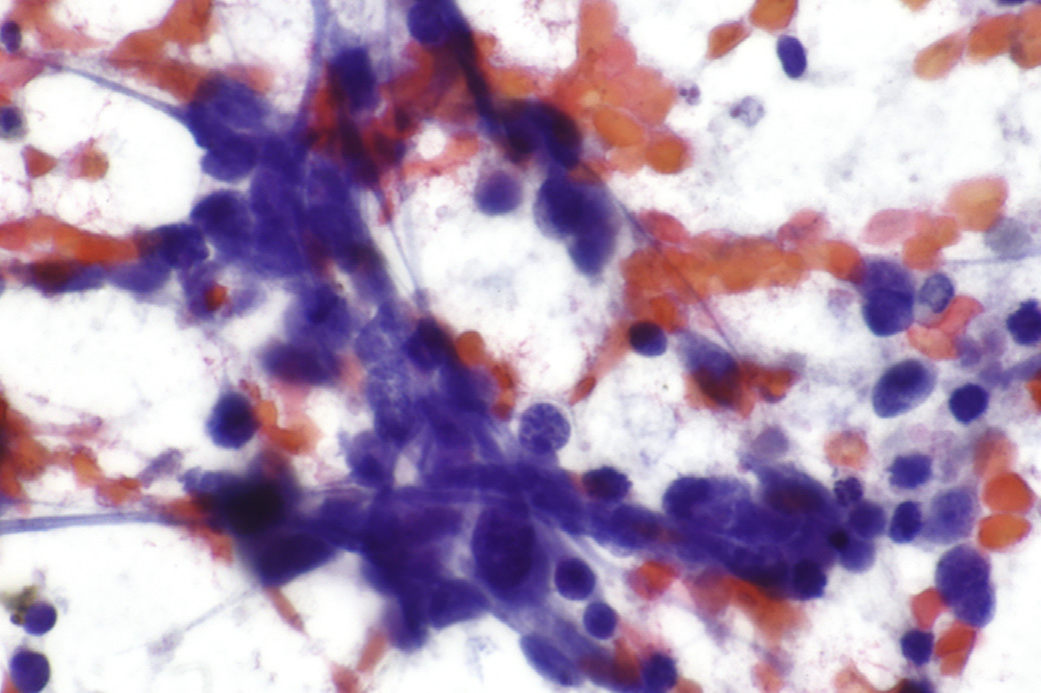
Cytological examination shows small-cell carcinoma cells with characteristic stippled chromatin. (Papanicolaou stain, x100) Image By Nephron – Own work, CC BY-SA 3.0, https://commons.wikimedia.org/w/index.php?curid=48340668. Republished under the Creative Commons Attribution (CC-BY-SA 3.0).

CT of the chest shows a 2.5 ​cm right pulmonary hilar mass and a 4 ​cm subcarinal mass. Magnetic resonance imaging of the brain, CT of the abdomen, and combined PET–CT scan of the skeleton are negative for metastases. Bronchoscopy with endobronchial brushing and lavage does not reveal any malignant cells. Fine needle aspiration of the subcarinal mass reveals the findings shown in Fig. 1, Fig. 2. Additional histologic testing utilizing various cell markers and antibodies confirms the diagnosis of small-cell carcinoma.

## Questions/discussion points, Part 3

### Why did the chest x-ray eight months prior not reveal the lung cancer?

Plain chest radiographs are insensitive for the detection of small early lung cancers. Chest x-ray is less than one-third as sensitive as CT for the detection of lung cancer.^13^ So, it should be no surprise that chest radiograph eight months prior failed to reveal the tumor that was presumably present.

### Why was bronchoscopy negative?

Small-cell lung carcinoma is typically central, arising in a proximal airway location, but bronchoscopy was negative in this case because small-cell carcinoma of the lung is typically submucosal, underneath the mucosa, so brushing and washing of the mucosa does not reveal it.^14^ In contrast, squamous cell carcinoma of the lung typically produces an endobronchial mass in a proximal airway, so that bronchoscopic cytology can yield the diagnosis. Adenocarcinoma of the lung is typically located in the periphery and very often not accessible by bronchoscopy, so it frequently requires transthoracic needle cytology or biopsy to make the diagnosis.

### What is the therapy for Lambert–Eaton syndrome?

Amifampridine is an oral medication that blocks voltage-gated potassium channels on the presynaptic neuron preventing the efflux of potassium ions, thus prolonging depolarization. This prolongs calcium influx, in turn increasing ACh release and improving neuromuscular function.^15^ Amifampridine significantly increases average compound muscle action potential (CMAP) amplitudes and significantly lowers neurological disability scores.^15^ The US Food and Drug Administration (FDA) approved amifampridine for treating Lambert–Eaton syndrome in November 2018 and seems destined to become first-line therapy for Lambert–Eaton myasthenia.^15^

Pyridostigmine is an oral medication that inhibits acetylcholinesterase, prolonging the presence of Ach in the synapse at the motor end-plate and thus improving neuromuscular function.^16^ It has long been used in the treatment of myasthenia gravis. Pyridostigmine is an alternative to amifampridine in the treatment of Lambert–Eaton myasthenia.^17^ If the neuromuscular disease is refractory to amifampridine and pyridostigmine, immunomodulatory therapy can be used. The first line immuno-modulatory therapy for Lambert–Eaton syndrome is intravenous immunoglobulin. Alternatives include prednisone, rituximab, azathioprine, or plasma exchange.^7^

### What is the prognosis for patients with Lambert–Eaton syndrome?

Prognosis for the neuromuscular disease of Lambert–Eaton syndrome by itself is good.^18^^,^^19^ A majority of patients remain or become independent for self-care after treatment and experience a stable disease course.^18^ Patients with Lambert–Eaton syndrome unassociated with tumor have normal survival. In sharp contrast, patients with Lambert–Eaton syndrome associated with small-cell lung carcinoma have survival determined by the tumor, and the survival of patients of patients with small-cell carcinoma remains poor.

## Teaching points

• The differential diagnosis of proximal weakness in the legs includes hypokalemia, dermatomyositis, polymyositis, immune-mediated necrotizing myopathy, statin therapy, alcoholic myopathy, thyroid myopathy, myasthenia gravis, and Lambert–Eaton syndrome.
• Lambert–Eaton syndrome has a classic triad of clinical manifestations: proximal muscle weakness, decreased tendon reflexes, and autonomic dysfunction, almost invariably with initial symptoms of leg weakness.
• Lambert–Eaton syndrome is an immunologic antibody-mediated disease from antibodies to voltage-gated calcium channels on presynaptic nerve terminals at the motor end-plate, which impair release of the neurotransmitter acetylcholine.
• Myasthenia gravis is an immunologic antibody-mediated disease from antibodies to postsynaptic acetylcholine receptors on the other side of the neuromuscular junction from Lambert–Eaton syndrome.
• Myasthenia gravis typically presents with oculobulbar weakness, without decreased deep tendon reflexes, and without associated autonomic symptoms.
• Lambert–Eaton syndrome superficially resembles a type II hypersensitivity reaction but lacks the cytotoxicity, complement activation or phagocytosis that characterizes type II reactions.
• Lambert–Eaton syndrome is often paraneoplastic, associated with small-cell lung carcinoma, and may become evident before the associated cancer, then making a search for the cancer appropriate.
• Lambert–Eaton syndrome is diagnosed by nerve conduction tests showing reduced compound muscle action potentials and serological tests showing antibodies to voltage-gated calcium channels.
• Lambert–Eaton syndrome can be treated with amifampridine, which blocks voltage-gated potassium channels on the presynaptic neuron, prolonging depolarization, calcium influx and acetylcholine release, thus improving neuromuscular function.

## Funding

The article processing fee for this article was funded by an open Award given by the Society of ‘67, which supports the mission of the 10.13039/100016205Association of Pathology Chairs to produce the next generation of outstanding investigators and educational scholars in the field of pathology. This award helps to promote the publication of high-quality original scholarship in *Academic Pathology* by authors at an early stage of academic development.
